# Comparison Benefit between Hydrogen Peroxide and Adrenaline in Tonsillectomy: A Randomized Controlled Study

**DOI:** 10.3390/jcm11102723

**Published:** 2022-05-11

**Authors:** Cheng-Yu Hsieh, Chuan-Jen Hsu, Hung-Pin Wu, Chuan-Hung Sun

**Affiliations:** 1 Department of Otolaryngology, Head and Neck Surgery, Taichung Tzu Chi Hospital, Buddhist Tzu Chi Medical Foundation, Taichung 427, Taiwan; chengyu384650@gmail.com (C.-Y.H.); cjhsu@ntu.edu.tw (C.-J.H.); hungpin_wu@yahoo.com.tw (H.-P.W.); 2 School of Medicine, Tzu Chi University, Hualien 970, Taiwan

**Keywords:** hydrogen peroxide, adrenaline, blood loss, tonsillectomy

## Abstract

This study aimed to further evaluate the benefit of topical hemostasis agents in tonsillectomy. We compared the clinical effects of topical application between hydrogen peroxide and adrenaline in tonsillectomy. Overall, 60 patients (120 tonsils) were prospectively enrolled for tonsillectomy between February 2018 and December 2020. The patients were randomly assigned to either the hydrogen peroxide or adrenaline group. Then, tonsillectomy was performed using hydrogen peroxide as a hemostatic agent on the assigned side, while adrenaline was applied to the other side. All procedures were performed by a surgeon who was blinded to the randomization. The outcome measurements of operation time, intraoperative blood loss, postoperative pain, and hemorrhage events were analyzed. The intraoperative blood loss was significantly lower in the hydrogen peroxide group than in the adrenaline group (9.99 ± 4.51 mL vs. 13.87 ± 6.32 mL; *p* = 0.0). The median operation time was also significantly lower in the hydrogen peroxide group (8.02 ± 3.59 min vs. 9.22 ± 3.88 min; *p* = 0.019). Meanwhile, the visual analogue scale (VAS) scores were significantly higher in the hydrogen peroxide group (4.98 ± 1.94 vs. 4.27 ± 1.97; *p* = 0.001). The topical application of hydrogen peroxide as a hemostatic agent effectively decreases the operation time and intraoperative blood loss. Thus, hydrogen peroxide can be used as a routine hemostatic agent for bleeding control in tonsillectomy.

## 1. Introduction

Tonsillectomy is one of the most common surgical procedures in otolaryngology. Despite improvements in anesthesia and surgical techniques, intraoperative and postoperative hemorrhage remain major concerns in tonsillectomy [1], with primary (<24 h) postoperative bleeding occurring in 0.3–5.4% of patients [2,3]. Primary postoperative bleeding is generally related to surgical techniques and hemostasis strategies, while secondary bleeding is more likely related to surgical site infection or sloughing of the eschar covering the tonsillar fossa.

The blood supply of the tonsils mainly comes from the lingual and tonsillar branches of the facial artery. The pharyngobasilar fascia, which extends into the tonsils, covers the lateral surface of the tonsils. The complexity of the blood supply of the tonsil and the distanced and limited operation field increase the risk of massive intraoperative bleeding during tonsillectomy. Therefore, a rapid-onset hemostasis agent is essential to avoid major surgical complications. Effective hemostasis contributes to a lower operation time, better outcomes, and uneventful wound healing.

Traditional electrocauterization for hemostasis may create thermal injury and result in explosive vaporization, which would lead to severe damage to the surrounding tissue. Topical hemostatic agents help the surgeon to target bleeding sources and reduce tissue damage in non-bleeding regions. In this regard, several topical agents, such as hydrogen peroxide, adrenaline, saline solution, and lidocaine [4,5,6], have been introduced to minimize blood loss. However, there is still no gold standard for topical hemostasis in tonsillectomy.

Post-tonsillectomy pain is another major problem, as it might lead to poor oral intake, dehydration, sleep disturbance, and prolonged hospitalization. Thus, the effect of pain control should be considered when a hemostasis agent is applied. Many local applications, such as bismuth sulfate, oral rinse, lidocaine spray, fibrin glue, and betadine silver nitrate, have been investigated to control postoperative pain [7].

Hydrogen peroxide is an oxidizing agent that is easily degraded by tissue catalase to form oxygen and water. It is a widely available topical antiseptic and nontoxic hemostasis agent that produces oxidative burst and local oxygen production [8]. In the early stages, the “bubble effect” may provide some chemical burn and mechanical debridement in areas of the wound that are not easily accessible to the surgeon. In addition, the bubble effect caused by erythrocyte catalase degradation of hydrogen peroxide can help the surgeon to localize areas that require cauterization and rapidly reduce hemorrhage [9]. In the late stages, delivering hydrogen peroxide into wounds can kill fibroblasts and promote re-epithelialization [10]. A previous report showed that the topical application of hydrogen peroxide could control hemostasis and greatly reduce operation time in tonsillectomy [5]. Adrenaline has also been demonstrated to be a reasonable hemostatic agent because of its low cost, low risk, powerful vasoconstrictor, and platelet aggregation. Topical use of adrenaline is an effective and reasonable hemostatic agent in tonsillectomy [11].

The advantages of both hydrogen peroxide and adrenaline include rapid onset, acceptable duration, easy accessibility, and cost effectiveness. However, to date, there has been no direct, comparative, randomized controlled trial to achieve consensus on the optimal topical hemostasis agent in tonsillectomy. Therefore, this study aimed to compare the clinical effects of the topical application of hydrogen peroxide and adrenaline in tonsillectomy.

## 2. Materials and Methods

### 2.1. Experimental Design

A total of 60 patients aged 8–68 years were prospectively enrolled for tonsillectomy in tertiary referral centers between February 2018 and December 2020. All subjects fulfilled the American Academy of Otolaryngology Head and Neck Surgery criteria for chronic or recurrent tonsillitis, recurrent tonsil hemorrhage, peritonsillar abscess, or tonsillar hypertrophy with obstructive symptoms. Subjects were excluded if they had tonsillar cancer, underwent combination surgeries, had severe underlying diseases, such as cardiovascular disease, or had bleeding tendency disorder.

All surgical procedures were performed via blunt dissection under general anesthesia by the same surgeon. The application of hydrogen peroxide and adrenaline was randomized preoperatively. A local anesthetic injection of 2 cc lidocaine over the peritonsillar area was performed prior to removing each side of the tonsil to reduce pain by blocking peripheral nociceptive excitation. To achieve the best confounding control, we focused on the same subjects, and all patients’ tonsils were randomly assigned to either the hydrogen peroxide or adrenaline group. Then, tonsillectomy was performed using hydrogen peroxide as a hemostatic agent on the assigned side, while adrenaline was applied to the other side. All procedures were performed by a “blind” surgeon. During tonsillectomy, cotton balls soaked with 3% hydrogen peroxide were tightly packed into the tonsillar fossa for hemostasis of mucosal bleeding, and 1% adrenaline was applied on the other side of the tonsillar fossa. We rinsed both cotton balls with 2% lidocaine and then packed them into the tonsillar fossa until complete hemostasis was achieved. Bipolar electrocauterization was used for hemostasis if persistent active bleeding was not controlled.

The intraoperative blood loss on each side was measured by weighing the cotton balls and suction bottle before and after the operation. The operation time was calculated as the period between the first incision and the time all bleeding or oozing was secured entirely on the single side, encompassing the time of dissection and hemostasis. We avoided opioid drugs due to nausea and possible respiratory inhibition. In addition, anti-inflammatory drugs, such as non-steroidal anti-inflammatory drugs, were excluded because of their adverse effects on platelet function, which are associated with a tendency to bleed.

Postoperative pain in the first 24 h and 48 h after tonsillectomy was recorded. Postoperative pain was assessed by determining the more painful side during follow-up. Pain intensity was evaluated by a blinded physician using a visual analogue scale, with a score of 0 indicating no pain and 10 indicating maximum pain. All patients were blinded to which technique was applied on each side. Postoperative data about pain score, fever, time to oral intake, and bleeding events were collected. All patients received the same dose of acetaminophen four times daily. In general, the patients were discharged 2 days postoperatively after examination of the uneventful surgical wound without oozing.

### 2.2. Statistical Analysis

Data on operation time, intraoperative blood loss, postoperative pain, and hemorrhage events were collected and analyzed. Descriptive statistics were presented as the means and standard deviations, and categorical variables were presented as counts and percentages. The 95% confidence intervals (CIs) were determined for the strength of association and intergroup correlation. For the main analysis, the differences in operation time, blood loss and postoperative pain between groups are expressed as means (95% CI). The study had a statistical power of 80% and an effect size of 70%. The paired *t* test was used to analyze postoperative pain score, intraoperative blood loss, and operation time. All statistical analyses were performed using SPSS 20.0 statistical software. *p* < 0.05 was considered statistically significant.

## 3. Results

### 3.1. Patient Characteristics

In total, 60 subjects were enrolled. None of the patients had any hypersensitivity response to the ingredients of the locally applied hydrogen peroxide and adrenaline. No complications or postoperative secondary bleeding were noted after tonsillectomy. The operation time, hemostasis time and intraoperative blood loss for each side are shown in Table 1. The postoperative pain scores in the first 24 h and 48 h after tonsillectomy are shown in Table 2. A comparison of the operation time, hemostasis time and blood loss on each side is shown in Table 3.

### 3.2. Outcomes

#### 3.2.1. Intraoperative Blood Loss

The average intraoperative blood loss was significantly higher in the hydrogen peroxide group than in the adrenaline group (9.99 ± 4.51 mL vs. 13.87 ± 6.32 mL, *p* = 0; Table 1). The ratio of patients with <10 cc blood loss was also significantly higher in the hydrogen peroxide group (61.6% vs. 36.6%).

#### 3.2.2. Operation Time

The median operation time was 8.02 ± 3.59 min in the hydrogen peroxide group and 9.22 ± 3.88 min in the adrenaline group (Table 1). Apparently, surgery was significantly faster in the hydrogen peroxide group than in the adrenaline group (*p* = 0.019; Table 1). The hemostasis time was also significantly shorter in the hydrogen peroxide group (3.43 ± 2.75 min vs. 4.49 ± 3.35 min, *p* = 0.07; Table 1).

#### 3.2.3. Postoperative Pain

The mean 24 h postoperative VAS score was significantly higher in the hydrogen peroxide group than in the adrenaline group (4.98 ± 1.94 vs. 4.27 ± 1.97, *p* = 0.001; Table 2). However, there was no significant difference in the mean 48 h postoperative VAS score between the two groups (3.47 ± 1.58 vs. 3.23 ± 1.52, *p* = 0.147; Table 2).

#### 3.2.4. Left versus Right Side Outcomes

The median operation time was 8.85 ± 4.04 min in the left group and 8.39 ± 3.49 min in the right group, with no significant difference (*p* = 0.458; Table 3). The median hemostasis time was 4.04 ± 3.23 min in the left group and 3.88 ± 2.99 min in the right group. The intraoperative blood loss in the left and right groups were 12.55 ± 6.28 and 11.30 ± 5.26, respectively, with no significant difference (*p* = 0.239).

## 4. Discussion

Post-tonsillectomy hemorrhage and pain are the major complications of tonsillectomy; the optimal modality for achieving hemostasis remains unclear. According to our results, both hydrogen peroxide and adrenaline can help to reduce intraoperative blood loss; moreover, the intraoperative blood loss and the median operation time were significantly lower in the hydrogen peroxide group than in the adrenaline group. To the best of our knowledge, this is the first study to compare hydrogen peroxide and adrenaline as hemostatic agents for tonsillectomy.

Unlike other studies that divided the patients into two groups, the distinctive characteristic of our study was that we focused on the same subjects; all patients served as their own control because hydrogen peroxide and adrenaline were applied to the opposing sides of the tonsillar fossa. Therefore, confounding factors, such as underlying disease, age, sex, and tonsil size, can be excluded. A few outliers may cause a disproportionate effect on the statistical results because of the small amount of intraoperative blood loss in tonsillectomy. For example, the influence of surgeon handedness in tonsillectomy has not been examined in previous reports. To eliminate differences due to handedness, we compared the operation time and intraoperative blood loss on each side and further analyzed by type of agent (hydrogen peroxide and adrenaline) (Table 4 and Table 5). Our results revealed that hand preference did not influence the overall outcomes based on operation time and blood loss, as evidenced by the lack of significant differences between the two groups.

A 2017 meta-analysis revealed that the application of local anesthetic, either by infiltration or the topical method, could provide a modest reduction in post-tonsillectomy pain and hemorrhage [12]. The meta-analysis concluded that a preoperative local anesthetic injection is a valuable method for decreasing blood loss and surgical time. Another meta-analysis suggested that topical local anesthetics on swabs provide similar analgesic effects as preoperative infiltration [13]. Previous studies showed that the general operation time by blunt dissection in tonsillectomy was 24.6–29.1 min [14,15]. Adopting the above-mentioned strategies, including preoperative local anesthetic injection and postoperative topical application of hemostatic agents, reduced the mean operation time to 9.99–13.87 min in our study.

Electrocauterization for hemostasis can significantly decrease the operation time and intraoperative blood loss; however, it can also increase postoperative pain [16,17]. Further, it also results in excessive eschar on the tonsillar fossa, which may cause secondary bleeding [3] and infection. In addition, the time to wound healing and return to a full diet is longer in patients undergoing bipolar cauterization hemostasis [18].

In our study, the intraoperative blood loss was small (median volume < 15 mL) in both the hydrogen peroxide and adrenaline groups. Topical hemostatic agents that have the benefit of rapid onset, easy accessibility, cost effectiveness and analgesic effect are highly beneficial. We performed blunt dissection and applied topical hemostatic agents. Topical application of a hemostatic agent can treat all potential bleeding sites, not only focusing on an active bleeding area, but also on hard-to-access bleeding areas, such as the low pole of the tonsil. Thus, a topical hemostatic agent may be a feasible method to control hemorrhage. Hemostasis with the compression of a cotton ball may also cause lower postoperative pain than bipolar cauterization and ligation [19]. Topical hemostatic agents can also prevent sloughing of the eschar and help control mucosal bleeding across surface areas. No secondary bleeding after tonsillectomy occurred in the present study.

Hydrogen peroxide is widely used for wound irrigation, owing to its hemostatic and antimicrobial effects. Chang et al. and Al-Abbasi et al. reported that the use of hydrogen peroxide significantly reduced the operation time in tonsillectomy by 35% and 31%, respectively [5,20]. In our study, hydrogen peroxide better reduced the operation time by 14.9% and achieved a better hemostatic effect than adrenaline. The decreased operation time in the hydrogen peroxide group could be due to the relatively short hemostasis time, in line with previous findings [5,20].

For intraoperative blood loss, the median volume was significantly lower in the hydrogen peroxide group than in the adrenaline group. We found that both hydrogen peroxide and adrenaline could decrease intraoperative hemorrhage. However, although the effect size of 3.88 mL of intraoperative blood loss may be significantly different, this little change may not have clinical significance. In addition, we also found that the mucosa and soft tissue turned white after hydrogen peroxide was pressed tightly. The chemical burns and bitter taste of hydrogen peroxide might explain the higher 24 h postoperative pain score in the hydrogen peroxide group (4.98 ± 1.94) than in the adrenaline group (4.27 ± 1.97).

There are three main applications of hydrogen peroxide: antiseptic, hemostasis and wound healing. Reactive oxygen species (ROS) defend the host from invading microbes by damaging microbial DNA. When hydrogen peroxide is degraded, reactive oxygen species are released, causing DNA strand breakage by DNA oxidization [21]. ROS induce interferon activation and result in an antiviral state, which limits viral replication. ROS may help promote cytokine production, autophagy and granuloma formation, resulting in an antimycobacterial state. By decreasing the colonization of bacteria and viruses, the severity of infection and pain can be reduced.

In addition to the antiseptic benefit, we also found a decrease in operation time. Further analysis of the decreased operation time in the hydrogen peroxide group showed that the “bubble effect”, due to oxidation in the early stage, rapidly turned the bleeding area to white. This helped the surgeon to easily localize the bleeding source requiring cauterization and clarify the visual field. It also shortened the operation time. Applying hydrogen peroxide to the wound at the late stage can kill fibroblasts and promote re-epithelialization [22]. Hydrogen peroxide facilitates hemostasis through several mechanisms, including platelet aggregation, stimulation of platelet-derived growth factor activation, and regulation of the contractility and barrier function of endothelial cells [23].

There are numerous theories regarding the hemostatic effects of hydrogen peroxide, including thermal injury of the vascular ends, oxygen embolization of vessels, and reactive vascular spasms [24]. More recently, it has been suggested that thrombolytic hyperactivity and thrombus formation can trigger hemostatic effects [24]. In addition, when catalase in red blood cells reacts with hydrogen peroxide, the chemical reaction induces the release of oxygen and heat, helping the surgeon to localize the bleeding site.

Currently, hydrogen peroxide is used clinically not only as a hemostatic and antiseptic agent, but also as a wound-healing agent [23]. Hydrogen peroxide may help to clear pathogen debris and promote cytokine secretion, helping tissue regeneration [25]. In our study, 3% hydrogen peroxide appeared to have no negative effect on wound healing. It should be noted that highly concentrated hydrogen peroxide (30%) carries a risk of cardiac arrest and stroke, due to oxygen embolism formation [26]. However, the use of a low concentration of hydrogen peroxide (3%) does not induce serious systemic side effects [9,24,27]. The application time should be limited to prevent tissue damage and limit pain. Collectively, these findings support the fact that 3% hydrogen peroxide is a safe and effective agent for intraoperative hemostasis and wound cleaning.

Hatton et al. reported that topical adrenaline is an effective hemostatic agent in tonsillectomy [11]. The application of bismuth subgallate and adrenaline paste to the tonsillar fossae reduced the operating time by 23% and blood loss by 21% [28]. Epinephrine, a platelet-stimulating agent, can cause aggregation of human platelets through alpha-adrenergic mechanisms [29]. In this study, we found that the topical use of adrenaline is mildly inferior to hydrogen peroxide, with respect to hemostatic function. The vasoconstriction effect of adrenaline on arterioles, capillaries and venules helps to delay intraoperative bleeding initially. However, post-tonsillectomy bleeding may result from a blood vessel that initially spasms and later resumes bleeding if hemostasis is not complete. Importantly, adrenaline takes longer to work in these cases. In the current study, the operation time and intraoperative blood loss were lower, at 14.9% and 38.8% (3.88 cc), respectively, in the hydrogen peroxide group than in the adrenaline group. However, in comparison with hydrogen peroxide, adrenaline was more effective in controlling postoperative pain in the first 24 h, but the pain scores were similar at 48 h postoperatively.

We combined lidocaine and adrenaline in this study because lidocaine could stabilize the neural membrane by inhibiting voltage-gated sodium channels, resulting in the suppression of impulse conduction, affecting local anesthetic action. To prevent the systemic circulation and adverse effects of central nervous system toxicity, tachycardia, convulsion, respiratory obstruction [30] and vocal palsy [31], adrenaline was applied topically. The vasoconstrictor property of adrenaline prolongs anesthesia activity and minimizes the risk of systemic circulation. By stimulating α-adrenergic receptors on the neural vasculature, combining adrenaline with lidocaine can lower the local blood flow, slow the clearance of lidocaine, and extend the duration of peripheral nerve block action. To eliminate differences in the pain control effect of the topical use of lidocaine, cotton balls rinsed with lidocaine were applied on both sides as the final step in the study. However, although rare, toxicity at high doses of lidocaine can influence cardiovascular and central nervous system function in a concentration-dependent manner.

This study has some limitations. The number of subjects enrolled in our study was too small to draw a definite conclusion. In the absence of a control group in this study, where no topical agents are used, it is difficult to interpret the absolute benefit of each other’s hemostatic agents. Previous studies measured pain before and after the administration of supplemental analgesia; however, there may still have been some residual analgesic effect on subsequent measurements in the early period. Meanwhile, we assessed the pain score at 24 h postoperatively, when the anesthetic effect may have little residual activity. Furthermore, we found that it was difficult for some patients to precisely discriminate the exact pain score on each side, possibly resulting in bias. Further studies should investigate the effects of hemostatic agents over a longer duration with a larger set of participants.

## 5. Conclusions

The topical application of hydrogen peroxide is beneficial for reducing the operation time and intraoperative blood loss, with minor complications, in tonsillectomy. Thus, hydrogen peroxide can be used as a routine topical hemostatic agent in tonsillectomy. Meanwhile, the topical application of adrenaline provides significant pain relief on the first day compared to hydrogen peroxide.

## Figures and Tables

**Table 1:** Between-group comparison of operation time, hemostasis time and blood loss.

Variables	Hydrogen Peroxide Group (*n* = 60)	Adrenaline Group (*n* = 60)	Median Difference Mean (95% CI)	*p*-Value *
Operation time (min)	8.02 ± 3.59	9.22 ± 3.88	−71.62 (−127.70, −15.53)	0.019
Hemostasis time (min)	3.43 ± 2.75	4.49 ± 3.35	−63.83 (−110.91, −16.76)	0.007
Blood loss (mL)	9.99 ± 4.51	13.87 ± 6.32	−3.88 (−5.76, −2.00)	0

Data are presented as the median values and 95% confidence intervals. The paired *t* test is used for continuous variables. * *p* < 0.05.

**Table 2:** Between-group comparison of postoperative pain score.

Variables	Hydrogen Peroxide (*n* = 60)	Adrenaline (*n* = 60)	Median Difference Mean (95% CI)	*p*-Value *
VAS, 24 h	4.98 ± 1.94	4.27 ± 1.97	0.72 (0.32, 1.12)	0.001
VAS, 48 h	3.47 ± 1.58	3.23 ± 1.52	0.23 (−0.83, 0.55)	0.147

Data are presented as the median values and 95% confidence intervals. The paired *t* test is used for continuous variables. * *p* < 0.05.

**Table 3:** Comparison of operation time, hemostasis time and blood loss by side of application.

Variables	Left (*n* = 60)	Right (*n* = 60)	*p*-Value *
Operation time (min)	8.85 ± 4.04	8.39 ± 3.49	0.458
Hemostasis time (min)	4.04 ± 3.23	3.88 ± 2.99	0.504
Blood loss (mL)	12.55 ± 6.28	11.30 ± 5.26	0.239

The paired *t* test is used for continuous variables. * *p* < 0.05.

**Table 4:** Intergroup correlation of operation time, hemostasis time and blood loss by side in the hydrogen peroxide group.

Variables	Left (*n* = 30)	Right (*n* = 30)	*p-*Value *
Operation time (min)	9.72 ± 3.99	8.43 ± 3.74	0.201
Hemostasis time (min)	3.56 ± 2.53	3.65 ± 2.50	0.891
Blood loss (mL)	10.62 ± 4.60	9.96 ± 4.91	0.589

The independent *t* test is used for continuous variables. * *p* < 0.05.

**Table 5:** Intergroup correlation of operation time, hemostasis time and blood loss by side in the adrenaline group.

Variables	Left (*n* = 30)	Right (*n* = 30)	*p-*Value *
Operation time (min)	7.99 ± 3.98	8.36 ± 3.29	0.696
Hemostasis time (min)	4.51 ± 3.79	4.12 ± 3.45	0.674
Blood loss (mL)	14.48 ± 7.18	12.64 ± 5.33	0.263

The independent *t* test is used for continuous variables. * *p* < 0.05.

## Data Availability

The data presented in this study are available on request from the corresponding author. The data are not publicly available due to privacy or research ethics.
